# Forest Bird Abundance Is Linked to the Interactions Between Forest Characteristics and Species Ecological Traits: Implications for Forest Management

**DOI:** 10.1002/ece3.72784

**Published:** 2026-01-19

**Authors:** Filip Szarvas, Jan Michálek, Zdeněk Vermouzek, Petr Voříšek, Jan Hanzelka, Jiří Reif

**Affiliations:** ^1^ Institute for Environmental Studies, Faculty of Science Charles University Prague Czechia; ^2^ T‐MAPY spol. s r.o Hradec Králové Czechia; ^3^ Czech Society for Ornithology Prague Czechia; ^4^ European Bird Census Council Prague Czechia; ^5^ Institute of Vertebrate Biology Czech Academy of Sciences Brno Czechia; ^6^ Department of Zoology, Faculty of Science Palacký University Olomouc Czechia

**Keywords:** abundance, birds, ecological niche, forest ecosystem, forest management, forestry, population trend

## Abstract

Majority of European forests are managed by intensive commercial forestry. At the same time, these forests are inhabited by numerous bird species expressing considerable diversity of ecological traits. From this perspective, it is important to learn how forest characteristics, resulting from modern forestry techniques, interact with ecological traits of forest bird species in shaping their abundance. To fill this knowledge gap, we used an extensive dataset on forest bird abundance collected within a national common bird monitoring scheme covering Czechia, a Central European country, supplemented by numerous variables describing forest stands collected within a national forest inventory and ecological traits of bird species extracted from literature. Our results show clear support for the interactions between bird traits and forest characteristics. Specifically, we found that migration distance emerged as the most important moderating bird trait interacting with all five forest characteristics, followed by habitat specialization, which interacted with four characteristics. From the management perspective, it is important to support forest stands with characteristics that are associated with the traits of species sharing unfavorable conservation status. This is the case for long‐distance migrants preferring broad‐leaved stands in lower growth phases and lower stem density, and habitat specialists preferring upland stands of higher growth phases and multiple vegetation layers. These two species groups can benefit from introducing specific management types, for example, coppicing in lowland areas for long‐distance migrants or protection of old‐growth stands in upland areas for habitat specialists.

## Graphical abstract

Using extensive bird monitoring data from Czechia and detailed forest and species trait information, this study found strong interactions between bird traits—especially migration distance and habitat specialization—and forest characteristics. These findings highlight the need for targeted forest management strategies, such as coppicing or old‐growth stand protection, to support species of conservation concern.

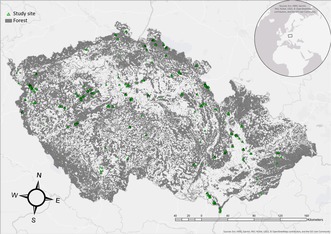

## 1 Introduction

Forests cover a substantial area of European land surface and are for the most part managed for timber production (Hansen et al. 2013). Despite their long history of intensive human use, forests still support species‐rich communities, which are inevitably influenced by human activities (Wang et al. 2022; Winkel et al. 2022). This is particularly true for European birds, a large proportion of which are strongly dependent on forests (Storchová and Hořák 2018). It is therefore crucial to understand how modern forestry practices affect their abundance (Paillet et al. 2010). Several previous studies described relationships between forest bird abundance and various forest characteristics including variables characterizing stands from the perspective of modern forestry (Anderson and Shugart 1974; Dettmers et al. 2002; Bakermans et al. 2012; Basile et al. 2021). These studies greatly improved our knowledge of habitat selection in numerous species, and we were even able to draw some important patterns at the community level. For instance, the representation of coniferous versus broad‐leaved trees and the stand's growth phase are characteristics of particular interest to forest managers and conservationists since they are affecting the abundance of a particularly high number of forest bird species (Kebrle et al. 2021, 2023; Szarvas et al. 2023).

However, the species‐specific patterns offer only limited insights into bird‐habitat associations as it is difficult to generalize these findings. In fact, species are defined by, among other things, various ecological traits (Webb et al. 2010) and it is thus interesting to ask what are the traits that underpin a common response of species to respective forest characteristics (Renner et al. 2024). At the same time, knowledge of these underpinnings remains incomplete. Therefore, in this study, we aim to link the abundance of these species to the different forest types taking the interactions with the trait values into account. By that means, we were able to identify the traits that may underlie interspecific variability of responses to various types of forest.

We specifically focus on four traits, namely insectivory, migration distance, habitat specialization and population trend, that cover different aspects of forest bird ecology. We recognize that population trend does not represent an ecological trait sensu stricto, but rather a species‐level characteristic. Nevertheless, because it is strongly influenced by species' ecological traits (e.g., Storch et al. 2023), we considered it relevant to include it here. Insects are an important source of proteins for birds and essential to facilitate nestling growth in many species (Getman‐Pickering et al. 2023; Schillé et al. 2024). Although insects are generally abundant during the breeding season of temperate forest birds (Reif et al. 2016; Evans et al. 2024), forest types differ in insect food availability for birds (Both et al. 2010), which may translate into varying abundance of species with different degrees of insectivory. Migration is a key aspect of birds' annual cycle whereby species track their resources in space, sometimes over long distances (Zurell et al. 2018). At the same time, species greatly differ in these distances when some of them remain in their territories throughout the year, whereas some others regularly cross entire continents or oceans (Berthold 2001). Migratory behavior is linked with some morphological adaptations (e.g., relatively longer wings) and phenology (e.g., breeding later in the season) (Newton 2023). We expect that these adaptations may be also linked to habitat associations at the breeding grounds when species migrating for longer distances may prefer sparser stands (being more permeable for their movement with relatively longer wings) (Norberg 1979) or stands where food is available later in spring. Habitat specialization enables species to exploit resources with a high efficiency—more closely a species is specialized to a given habitat, the higher is its abundance in that habitat (Devictor et al. 2010). In contrast, the abundance of generalist species does not vary substantially across habitats (Devictor et al. 2010). As human‐induced land cover changes typically favor the generalist over the specialist ones (Reif et al. 2013), it is important to learn which forest characteristics are associated with a higher habitat specialization of forest birds. Population trend measures the magnitude of population decrease or increase over a given period in all biotopes, not only forests (Wauchope et al. 2021). Ultimately, if a species' population continues to decline, its abundance may reach a critical threshold, and the species becomes extinct. In contrast, increasing trends indicate improving environmental conditions leading to a higher carrying capacity and hence higher abundance. Although population dynamics are driven by numerous factors, habitat often plays a key role in shaping population trends (Storch et al. 2023). Therefore, from a conservation perspective, it is essential to find the forest habitats characteristics associated with the occurrence of species with decreasing and increasing trends.

In this study, we focus on the following forest characteristics that are widely used in management practice in central Europe: growth phase, proportion of coniferous trees, number of vegetation layers, stem density, and altitude. The growth phase describes the appearance of the stand, discriminating young, shrub‐like dense forest from the mature forest with a high canopy and rich tree cavities (Poleno et al. 2009). According to some studies, the distinction between dense shrub‐like stands and mature stands with a high canopy is the most important factor determining bird community composition in temperate forests (Kameniar et al. 2021). The proportion of coniferous trees reports on the representation of coniferous vs. broad‐leaved trees in a given stand. Bird preference for one of these tree types is linked with numerous foraging, breeding, and behavioral adaptations, so it is unsurprising that this characteristic is important for many bird species (Szarvas et al. 2023). In Czechia, the number of vegetation layers and stem density serve as descriptors of the structural complexity of stands. According to these characteristics, one can discriminate forests of seminatural appearance, that is, those with more vegetation layers and lower stem density, from intensively managed forests with only one vegetation layer and a higher stem density (Poleno et al. 2009). Therefore, potential relationships of bird species to these characteristics are informative in respect of their sensitivity to management intensity.

Here we aim to relate the abundance of respective bird species to their ecological traits in the interaction of the above‐mentioned forest characteristics. By that means, we will uncover what the forest types linked to specific trait values are. Knowledge of these relationships can be used to make informed management decisions.

## 2 Methods

### 2.1 Study Area

The study covers the entire Czechia as it is based on a national citizen science monitoring project (see Bird data below). Czechia is a central European country with an altitudinal span from the lowland areas below 200 m asl to mountain areas above 1000 m asl, but most of the country's area is covered by mid altitudes (Chytrý et al. 2001). The vast majority of Czech forests are managed for timber production (MZe 2021). They are even‐aged monocultures, dominated by Norway spruce ( *Picea abies* ) in the mid to higher altitudes and by Hornbeam ( *Carpinus betulus* )/Oak ( *Quercus robur* ) and Scotts pine ( *Pinus sylvestris* ) in the lowlands, while Beech ( *Fagus sylvatica* ) dominates higher altitude natural forest vegetation (MZe 2021). Coniferous trees account for 70% of all trees planted in Czechia and most of the coniferous stands are situated outside of the range of ecological conditions in which the tree species (mainly Norway spruce) constituting these stands naturally occur (MZe 2021). Montane forests still show signs of anthropogenic damage caused by acid rains resulting from emissions of sulfur dioxide that occurred in the 1970s–1990s, but the forests started regeneration at the beginning of the 21st century (Flousek 2000; Vacek et al. 2015).

### 2.2 Bird Count Data

Bird count data were collected within the Breeding Bird Monitoring Program (in Czech “Jednotný program sčítání ptáků”—JPSP) organized by the Czech Society for Ornithology (Reif et al. 2013). JSPS is based on annual counts of common breeding bird species covering the whole area of Czechia. Counts are conducted by experienced volunteer ornithologists on 350 transects under a standardized protocol. Each transect consists of exactly 20 counting points separated by 300 to 500 m from each other. During a 5‐min count performed on a given point under favorable weather conditions (no heavy rain or strong wind), all seen and heard bird individuals are recorded. The points are visited twice per breeding season (April–June) to record both early and late breeders (Reif et al. 2007). For further analysis, we took the maximum count of a given species at each point recorded in a given year, assuming that it is closer to real abundance than the mean count (Godet et al. 2007). In addition, we used only the observations within a 100 m radius from a point to improve accuracy (Kéry and Schmid 2006; Kéry and Schmidt 2008).

**FIGURE 1:**
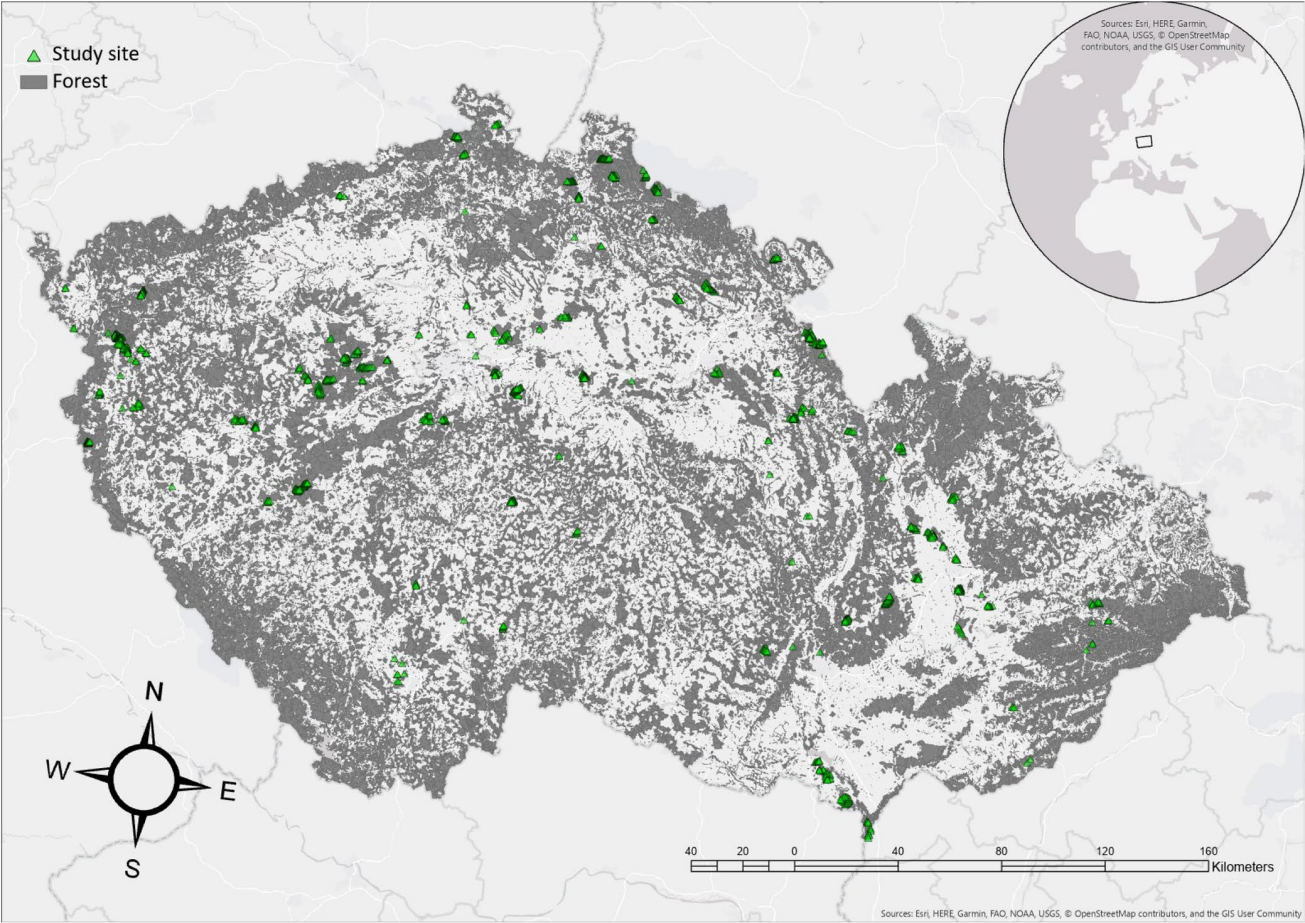
Map of sites counted within the Breeding Bird Monitoring Program in Czechia (green symbols).

To exclude possible influence of non‐forest habitats and edge effects, we used bird count data only from points where forest coverage was higher than 99% in 100 m radius (Szarvas et al. 2023). Forest coverage was calculated using Corine Land Cover database (European Environment Agency 2021). The final dataset included bird data from the points with desired forest coverage from years 2010–2020 where counts were carried out in at least 3 consecutive years during this time period, that is, data obtained from 983 census points on 107 transects (Szarvas et al. 2023) (Figure 1).

From this dataset, we excluded the species that are not reliably covered by the counting method developed for small territorial passerines, that is, colonial breeders, birds of prey, other large species etc. As a result, we used 43 species for further analysis (Table S1).

### 2.3 Forest Characteristics

For purposes of this study, we used four forest characteristics obtained from the National Forestry Institute (NFI)—Czech national authority responsible for collecting, calculating, and providing forest data in Czechia. Each variable was obtained for a single year within the period covering the bird data (see above). Although this seems like a temporal mismatch, annual changes are typically modest in forest ecosystems, so we can reasonably assume that the forest variable from a single year characterizes a given forest stand adequately for the entire period 2010–2020 (Poleno et al. 2009). From the spatial perspective, values of respective variables were expressed for the 100 m radius around each census point, that is, precisely matching the areas covered by bird counts. Growth phases are data provided by remote sensing; other variables come from Czech national forest inventory (2021).

Definitions of respective characteristics and processing of these data for purposes of the analysis are detailed in Szarvas et al. (2023). Here we briefly summarize the key information on each characteristic. *Growth phase* is recognized as four categories of stands: (i) glade/culture/seedling, forest/young growth/thicket—trees from seedling to trees with formed canopy up to 5 cm in diameter at breast height (DBH); (ii) small pole stand –of the DBH between 6 and 12 cm; (iii) pole timber stand—of the DBH between 13 and 19 cm; (iv) high forest—stand of trees of the DBH more than 20 cm and the age more than 50 years (Poleno et al. 2009). For the analyses, we calculated percentual coverage of the sum of latter two categories, that is, pole timber stand and high forest, as they represent habitat for birds of later successional stages, while the former two categories are occupied by birds of early successional stages (Szarvas et al. 2023). *Proportion of coniferous trees*. The data describe the percentage of coniferous and broad‐leaved trees present in each stand. Because these proportions are mutually exclusive (i.e., they sum to 100%), we used the percentage of conifers in the analysis. *Number of vegetation layers* is distinguished using three categories: (i) single layer—even aged monocultures; (ii) two layers—multi‐species stands in managed forests; (iii) three layers—mostly semi‐natural or natural stands (Poleno et al. 2009). As the number of vegetation layers at a given point we considered the category with the highest representation at that point. *Stem density* expresses the proportion of stand space filled by tree stems from 0% (no trees present in a given stand) up to 100% (all space theoretically available is filled by tree stems). Importantly, 100% stem density can mean both large number of thin trees, as well as smaller number very large trees. For the analysis, we calculated relative area of a census point covered by stands with stem density of 70% and higher. As an additional variable, we used *altitude* which was obtained from Czech geoprocessing institute (ČÚZK) (ČÚZK 2023).

### 2.4 Bird Species Traits

Data on four bird species traits (Table S1) that we considered for this study were obtained from literature, namely from Hanzelka et al. (2019), Reif and Hanzelka (2020), Reif et al. (2010), and Storch et al. (2023). Here we briefly characterize each trait: *insectivory* describes species dependence on insects in the diet across the whole year recognizing species that do not feed on any insects (trait value = 0) from partial (1) and full (2) insectivores. *Migration distance* is the geographic distance between centroids of species breeding and non‐breeding range. *Habitat specialization* was expressed using species specialization index (SSI) (Julliard et al. 2006) showing the lowest values in habitat generalists and the highest values in specialists. *Population trend* is the mean annual change in population of a given species over the period from 1982 to 2020 in the whole territory of Czechia based on fitting long‐linear models on national monitoring data.

### 2.5 Data Analysis

The analysis aimed to investigate how forest characteristics interact with species traits in shaping bird abundance. For this purpose, we fitted a phylogenetic generalized least square model (PGLS) including all species within a single analysis. Although the model was complex, this approach enabled a rigorous test of the interactive effects of species‐level traits on bird abundance. The model used a negative binomial error distribution, with the number of individuals of each species recorded at counting points in each year as the response variable, while forest characteristics, species traits, and their interactions were explanatory variables. These interactions were specified as two‐way interactions between explanatory variables, with each of the four bird traits interacting with each of the five forest characteristics (20 interactions in total). To account for non‐independence arising from repeated sampling, counting points nested within sites were included as random effects because each site consisted of 20 counting points. We also initially tested year as an additional random effect, but as it explained only a negligible proportion of variance, it was excluded from the model used for inference. In addition, to control for phylogenetic relatedness among species, we incorporated a phylogenetic structure into the model, thereby accounting for potential phylogenetic non‐independence in species‐level responses. For this purpose, we downloaded 100 trees from Jetz et al. (2012) and composed a consensus tree which we included among random effects. All explanatory variables were scaled to zero mean and unit variance prior to analysis. Model assumptions were evaluated using the DHARMa package (Hartig 2022), which indicated no violations. Collinearity diagnostics showed that no model term had a variance inflation factor above 0.46, indicating no concern regarding collinearity.

We used R version 4.4.1 (R Core Team 2024) for all analyses. Consensus tree was built using APE package (Paradis et al. 2004) and phytools package (Revell 2012). PGLS was run with the glmmTMB package (Brooks et al. 2023). Respective interactions were visualized using the package ggplot2 (Wickham 2011) and emmeans (Lenth 2024).

## 3 Results

Bird abundance was significantly shaped by several forest characteristics in their interactions with bird traits (Table 1). Specifically, migration distance showed significant interaction with all five forest characteristics, and habitat specialization, insectivory, and population trend with four forest characteristics (Table 1).

**TABLE 1:** Performance of respective explanatory variables (forest characteristics and bird species traits) and their interactions in explaining variability in counts of birds in Czech forests obtained from a generalized linear mixed model.

Model term	df	*χ* ^2^	*p*
Growth phase	1	26.80	0.000
Proportion of conifers	1	4.66	0.031
Number of vegetation layers	1	5.58	0.018
Stem density	1	5.07	0.024
Altitude	1	4.35	0.037
Insectivory	1	0.65	0.419
Migration distance	1	0.10	0.748
Habitat specialization	1	0.01	0.916
Population trend	1	8.47	0.003
Growth phase × insectivory	1	104.45	0.000
Growth phase × migration distance	1	664.37	0.000
Growth phase × habitat specialization	1	499.62	0.000
Growth phase × population trend	1	216.65	0.000
Proportion of conifers × insectivory	1	5.42	0.020
Proportion of conifers × migration distance	1	173.96	0.000
Proportion of conifers × habitat specialization	1	6.10	0.014
Proportion of conifers × population trend	1	526.04	0.000
Number vegetation layers × insectivory	1	3.26	0.071
Number vegetation layers × migration distance	1	22.29	0.000
Number vegetation layers × habitat specialization	1	11.80	0.000
Number vegetation layers × population trend	1	8.47	0.004
Stem density × insectivory	1	45.23	0.000
Stem density × migration distance	1	15.51	0.000
Stem density × habitat specialization	1	0.06	0.809
Stem density × population trend	1	2.71	0.100
Altitude × insectivory	1	410.89	0.000
Altitude × migration distance	1	12.43	0.000
Altitude × habitat specialization	1	873.29	0.000
Altitude × population trend	1	276.51	0.000

**FIGURE 2:**
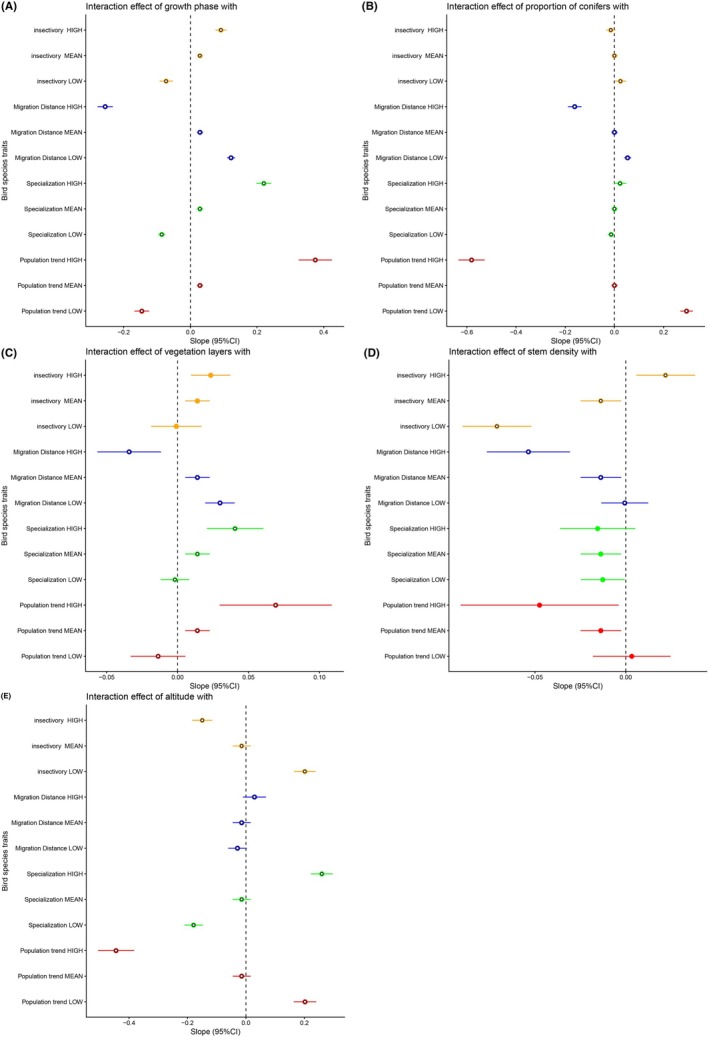
The relationships of bird counts in Czech forests to interactions between forest characteristics and bird species traits estimated by a generalized linear mixed model. Each plot shows two‐way interactions between respective bird species traits and (A) growth phase, (B) proportion of conifers, (C) number of vegetation layers, (D) stem density, and (E) altitude. Effects of respective interactions on bird counts are presented as regression slopes (dots) with 95% confidence intervals (CI, wiskers). To depict the range of effect expressed by a given interaction between a trait and forest characteristic, we calculated separate slopes for −1 * standard deviation (“low”), mean value (“mean”) and +1 * standard deviation (“high”) of each bird species trait. Significant interactions are depicted by open symbols.

Regarding migration distance, counts of birds migrating longer distances increased toward forest stands of lower growth phases (Figure 2A) and broad‐leaved trees (Figure 2B), whereas counts of species that migrate shorter distances or do not migrate at all showed the opposite pattern, that is, increased toward stands of higher growth phases (Figure 2A) and coniferous forests (Figure 2B). Longer migration distances were also associated with a steeper count increase with lower stem density than shorter migration distances (Figure 2D). On the other hand, counts of species migrating shorter distances increased toward forests with more vegetation layers (Figure 2C) and forests at higher altitudes (Figure 2E), while counts of species migrating longer distances did not (Figure 2C,E).

In respect to habitat specialization, counts of more specialized species increased toward forest stands of higher growth phases (Figure 2A), higher proportions of conifers (Figure 2B), more vegetation layers (Figure 2C), and located in higher altitudes (Figure 2E), while counts of less specialized species increased toward forests of the opposite characteristics, that is, being of lower growth phases (Figure 2A), with higher proportions of broad‐leaved trees (Figure 2B), fewer vegetation layers (Figure 2C) and in lower altitudes (Figure 2E). Stem density showed no significant interaction with habitat specialization (Figure 2D).

Counts of species that are more dependent on the insects in their diet increased toward forest stands of higher growth phases, whereas counts of species that are less dependent on the insects increased toward stands with lower growth phases (Figure 2A). Insectivores were also more abundant in stands with a higher proportion of broad‐leaved trees and higher stem density, and the opposite was found for non‐insectivores (Figure 2B,D). Counts of species that are less insectivorous increased toward higher altitudes, whereas counts of species that are more insectivorous decreased (Figure 2E). Number of vegetation layers (Figure 2C) showed no interaction with insectivory.

Counts of species with negative national population trends increased toward forest stands of lower growth phases (Figure 2A), a higher proportion of coniferous trees (Figure 2B), and locations at high altitudes (Figure 2E), while counts of species with positive population trends increased toward forest stands of higher growth phases (Figure 2A), a higher proportion of broad‐leaved trees (Figure 2B), more vegetation layers (Figure 2C), and locations at low altitudes (Figure 2E). Stem density did not show a significant interaction with population trend (Figure 2D).

## 4 Discussion

Our analysis of bird monitoring data collected at the national scale in Czechia showed a strong indication that abundance responses of bird species to various forest characteristics are moderated by bird traits. Our models showed support for the majority of interactions between forest characteristics and bird traits, where the proportion of conifers and stand growth phase had the strongest effects on bird abundance, depending on the values of particular bird traits. Below we discuss the individual patterns.

### 4.1 Migration Distance

Long‐distance migrants (e.g., Tree Pipit *Anthus trivialis* or Willow Warbler *Phylloscopus trochilus* ) prefer stands of lower growth phases, stands with a higher proportion of broad‐leaved trees, fewer vegetation layers and a lower stem density. One explanation for these preferences may lie in species' non‐breeding grounds which are located in sub‐Saharan Africa for the long‐distance migratory species in our dataset. Here the species spend the longest part of their annual cycle (Briedis et al. 2019). Although these species breed in forest, they winter in the Sahel or savanna zone where vegetation is sparse, that is, the stem density is low (Zwarts et al. 2023). In the wintering and stop‐over sites, they occupy bushy habitats, often formed by various acacia species, and vegetation on these sites resembles Central European broad‐leaved stands of lower growth phases (Atkinson et al. 2014). As the majority of Palearctic long‐distance migrants originated in their present‐day wintering grounds and spread northwards to their current breeding areas (Bruderer and Salewski 2008), we suggest that their breeding habitat preference to some extent conserved the vegetation structure and composition of the area of their origin. As niche conservatism is common in birds (Stukenholtz and Stevens 2024), we find the explanation plausible.

Species migrating for shorter distances or not migrating at all show opposite preferences in growth phase and proportion of coniferous trees. We suggest that these opposite preferences may partly result from interspecific competition which is quite common between species showing different migratory strategies such as Great Tit ( *Parus major* ) and European Pied Flycatcher ( *Ficedula hypoleuca* ) (Samplonius and Both 2017). To reduce the cost of utilizing the common resources, selection pressures act toward preferences of opposing ends of a given niche attribute (Pfennig and Pfennig 2009), that is, proportion of conifers and growth phase in this case. In the case of stem density, the species migrating for shorter distances showed a similar direction of the relationship as the long‐distance migrants, but with a shallower slope indicating that these species do not respond much to this characteristic.

In contrast, the abundance of short‐distance migrants and non‐migrants increases with the number of vegetation layers and altitude (e.g., Eurasian Bullfinch *Pyrrhula pyrrhula* or Black Woodpecker *Dryocopus martius* ), while the abundance of long‐distance migrants decreased. One explanation for the former pattern may be the necessity to exploit multiple layers to obtain enough food to survive harsh weather conditions during winter. Species like tits, which typically spend their whole annual cycle close to or at their breeding grounds, forage both in canopy, as well as in the shrub layer and near ground vegetation (Berlusconi et al. 2022), and we can speculate that this may be an adaptation to facilitate winter survival, and, therefore, they prefer multilayer stands for breeding, too. The effect of winter conditions may also explain why year‐round residents prefer coniferous trees—as most of these tree species are not deciduous, the presence of leaves during winter can support birds that search for food in this habitat. The effect of altitude is expectable from a macroecological perspective because the representation of short‐distance and resident species increases toward higher elevations (Scridel et al. 2018). It is possible that the ability to spend winter in the temperate zone enables the species to occupy more extreme conditions for breeding, while species migrating to the tropics prefer milder conditions with more abundant resources (Lemoine et al. 2007).

### 4.2 Habitat Specialization

More specialized bird species (e.g., Crested Tit *Lophophanes cristatus* or Red Crossbill *Loxia curvirostra* ) prefer stands of higher growth phases, dominated by coniferous trees, having multiple vegetation layers and being located at higher altitudes, while the generalists (e.g., Common Chiffchaff *Phylloscopus collybita* or Eurasian Jay *Garrlus glandarius*) show opposite preferences. The effects of growth phases and vegetation layers correspond with a general idea of the evolution of ecological specialization according to which this way of life is most beneficial in complex and stable environments (Sexton et al. 2017): vertically heterogeneous mature stands provide more ecological niches which can be occupied by numerous specialized species (Rigo et al. 2024). In contrast, homogeneous unstable environments are more frequently occupied by habitat generalists (Sexton et al. 2017). In our case, such environments can be represented by lower growth phases with a lower number of vegetation layers. These stands of younger trees are progressively aging, being rather unstable in space and time. Such a lack of stability may be linked to lower specialization of birds occupying these stands.

In the case of the effect of altitude, it concurs with the observation of selection for a high ecological specialization in mountain conditions, likely due to the ability to cope with a lower effective oxygen concentration and extreme climate (Rivas‐Salvador et al. 2019). Therefore, more specialized forest bird species are more abundant in forests at higher altitudes.

Regarding the tree species composition, the explanation is more complex. Under natural conditions, coniferous forests undergo large‐scale disturbance dynamics due to the impacts of windstorms and fire (Pickett and White 1985). Such disturbances select for specific sets of species occupying large, impacted areas (Puig‐Gironès et al. 2023), while species inhabiting high coniferous stands that avoid these areas are thus more specialized (Wesołowski et al. 2018). In broad‐leaved forests, disturbance dynamics are confined to smaller spatial scales (Pickett and White 1985), and canopy gaps created by these disturbances are tolerated by high‐stand species having thus broader niches (Wesołowski et al. 2018).

### 4.3 Insectivory

Species that are more dependent on the insects (e.g., Collared Flycatcher *Ficedula albicollis* ) prefer forests of higher growth phases and stem density, and with a higher share of broad‐leaved trees in contrast to species that are not insectivorous (e.g., Common Wood Pigeon *Columba palumbus* ). Stands of higher growth phases have more developed canopy and generally larger leaf area than stands of lower growth phases (Asner et al. 2003). Such a development of green vegetation creates a habitat for the insects being available as a food supply for insectivorous forest birds (Krištín and Patočka 1997). Moreover, higher growth phase stands are typically of older age which is connected with more dead wood hosting saproxylic insects (Jacobs et al. 2007). Greater insect habitat availability is also provided in stands of a higher stem density. By that means insectivorous bird species may benefit from such stands. Similarly, broad‐leaved tree species host more phytophagous insects than coniferous trees (Sierzega and Eichholz 2019; Kim and Choi 2021), which probably translated into preference of broad‐leaved stands by insectivores in our data.

In contrast, less insectivorous species prefer forests in higher altitudes. It can be explained by decreasing insect diversity and abundance toward higher elevations (Wolda 1987). Insects are ectothermic organisms whose growth is greatly limited by temperature, so their availability is inevitably higher in climatically more suitable lowland areas than in mountains. Therefore, to occupy higher elevations, birds must include alternative food resources in their diet, which inevitably decreases their degree of insectivory. So, we can say that even though the mountain birds are habitat specialists, as we describe above, they are diet generalists at the same time.

### 4.4 Population Trend

Species with decreasing population trend (e.g., Goldcrest *Regulus regulus* or Dunnock *Prunella modularis* ) prefer forest stands at high altitudes, of lower growth phases, lower number of vegetation layers and with a higher proportion of coniferous trees, whereas species whose national populations increase (e.g., Stock Dove *Columba oenas* or European Green Woodpecker *Picus viridis* ) prefer stands at low altitudes, of higher growth phases, dominated by broad‐leaved trees and more vegetation layers.

The effects of growth phase, vegetation layers and tree species composition broadly correspond with changes in composition of Czech forests that occurred over several last decades (Reif et al. 2022) acting in line with broad pan‐European trends (McGrath et al. 2015). These changes were mainly driven by a shift in the purpose of forest management from fuel production to timber harvesting, resulting in prolongation of rotation periods (from 15–30 years to 80–120 years) and stand maturation (Müllerová et al. 2015; Riedl et al. 2020). In addition, climatic changes accompanied by higher atmospheric CO_2_ concentrations and higher temperatures facilitate tree growth and higher wood stock (Schulze et al. 2019). Taken together, forests become more mature, providing more breeding opportunities for species preferring higher growth phases and more vegetation layers, whose populations increase, whereas suitable habitat area becomes smaller for species preferring lower growth phases and less complex stands, resulting in their population declines. Moreover, since the second half of the 20th century, tree species composition has been changing from coniferous to broad‐leaved species, even though the coniferous species, most notably Norway spruce, still dominate (Riedl et al. 2020). This change most likely resulted in changes in bird community composition, where populations of species associated with coniferous or broad‐leaved trees decline or increase, respectively.

The negative effect of altitude can be explained by the impacts of windstorms and bark‐beetle outbreaks whose higher frequency over the last two decades, induced by climate change, resulted in large‐scale dieback of mountain forests (Konopka et al. 2016; Netherer et al. 2019). Moreover, it could also contribute to negative trends in species preferring conifers as these are particularly impacted (Hlásny et al. 2021).

### 4.5 Management Recommendations

Based on our results, we can formulate the following recommendations for forest management in Central Europe, incl. the implementation of the EU Regulation 2024/1991 on nature restoration recently adopted by the EU:

1. As long‐distance migrants are experiencing severe population depletion across Europe (Vickery et al. 2023), we suggest that the stands with the characteristics preferred by these species should be supported at least in forests managed for conservation purposes. This includes decreasing stem density, planting mainly broad‐leaved tree species and focusing on lower growth phases. We suggest that these characteristics can be fulfilled, for instance, by restoration of coppicing and other ancient management approaches such as low forest and forest pasture (Vymazalová et al. 2021), which already occurs in several European regions (MacColl et al. 2014; Kamp 2022). On the other hand, these efforts may be insufficient since long‐distance migrants' populations are also influenced by changes on wintering grounds (Zwarts et al. 2023).
2. Similar to long‐distance migrants, habitat specialists are at risk of regional extinctions (Le Viol et al. 2012). To support their populations, we suggest that multilayer stands should be created, especially in higher altitudes which are preferred by these species (Cordeiro Pereira et al. 2024). We propose that this can be achieved by conservation of montane old‐growth forests and by providing conditions for natural regeneration instead of salvage logging following forest diebacks (Thorn et al. 2016)
3. As insectivorous species show the preference for stands of higher growth phases, we do not suggest any specific recommendation for their support because such stands are currently greatly preferred by commercial forest management across Europe (McGrath et al. 2015), and they can also benefit from climatic changes (Reif et al. 2022)
4. Forest bird species showing the negative population trends at the national scale are often those with the most abundant European populations due to their preference for coniferous trees that are widely planted (Burns et al. 2021), but their cover is generally decreasing. As this decrease is desirable from a conservation perspective because it leads to recovery of natural forest vegetation (Neuhäuslová et al. 2001) and national populations of these bird species are very large, we do not recommend taking actions to reverse their population trends. However, declining species showing a preference for lower growth phases should undoubtedly benefit from restoration of ancient forest management types we recommend in the first point if introduced on sufficient areas of forests.

## Author Contributions

**Filip Szarvas:** conceptualization (equal), data curation (equal), formal analysis (equal), visualization (equal), writing – original draft (equal). **Jan Michálek:** data curation (equal), writing – review and editing (equal). **Zdeněk Vermouzek:** data curation (equal), writing – review and editing (equal). **Petr Voříšek:** data curation (equal), writing – review and editing (equal). **Jan Hanzelka:** formal analysis (equal), visualization (equal), writing – review and editing (equal). **Jiří Reif:** funding acquisition (equal), supervision (equal), writing – original draft (equal).

## Conflicts of Interest

The authors declare no conflicts of interest.

## Supporting information


**Data S1:** ece372784‐sup‐0001‐DataS1.xlsx.

**Appendix S1:** ece372784‐sup‐0002‐AppendixS1.docx.

## Data Availability

All the required data are uploaded as Supporting Information.
