# Supplementary material for: Forest Bird Abundance Is Linked to the Interactions Between Forest Characteristics and Species Ecological Traits: Implications for Forest Management
Source: Ecol Evol. 2026 Jan 19;16(1):e72784. doi: 10.1002/ece3.72784 (PMC12815597; doi:10.1002/ece3.72784)
Supplement: Supplementary file 2 — Appendix S1: ece372784‐sup‐0002‐AppendixS1.docx. [file ECE3-16-e72784-s001.docx]

**Supplementary Table S1:** List of bird species considered in this study with their scientific names, common names, species abbreviations and mean abundance per count point.

| Scientific name | Common name | Species Abbreviation | Habitat specialization | Population trend | Dependence on insects | Migration distance (km) |
| --- | --- | --- | --- | --- | --- | --- |
| ***Anthus trivialis*** | **Tree Pipit** | Ant_tri | 0.7082 | -0.0345 | 2 | 6086 |
| ***Coccothraustes coccothraustes*** | **Hawfinch** | Coc_coc | 0.8013 | -0.0132 | 1 | 748 |
| ***Certhia brachydactyla*** | **Short-toed Treecreeper** | Cer_bra | 1.1992 | -0.01 | 2 | 0 |
| ***Certhia familiaris*** | **Eurasian Treecreeper** | Cer_fam | 0.873 | 0.0032 | 2 | 0 |
| ***Columba oenas*** | **Stock Dove** | Col_oen | 1.6807 | 0.0218 | 0 | 1541 |
| ***Columba palumbus*** | **Common Wood Pigeon** | Col_pal | 0.4167 | 0.0351 | 0 | 1701 |
| ***Corvus corax*** | **Common Raven** | Cor_cor | 0.5449 | 0.1071 | 1 | 11 |
| ***Curruca communis*** | **Common Whitethroat** | Syl_com | 0.7487 | 0.0025 | 1 | 5496 |
| ***Curruca curruca*** | **Lesser Whitethroat** | Cur_cur | 0.5762 | 0.0063 | 1 | 4056 |
| ***Cyanistes caeruleus*** | **Blue Tit** | Cya_cae | 0.3869 | 0.0107 | 1 | 5 |
| ***Dendrocopos major*** | **Great Spotted Woodpecker** | Den_maj | 0.7869 | 0.0158 | 1 | 0 |
| ***Dryocopus martius*** | **Black Woodpecker** | Dry_mar | 0.8967 | 0.0219 | 1 | 0 |
| ***Emberiza citrinella*** | **Yellowhammer** | Emb_cit | 0.6017 | -0.0107 | 1 | 382 |
| ***Erithacus rubecula*** | **European Robin** | Eri_rub | 0.8576 | 0.0105 | 1 | 1466 |
| ***Ficedula albicollis*** | **Collared Flycatcher** | Fic_alb | 1.6973 | 0.015 | 2 | 7114 |
| ***Ficedula hypoleuca*** | **European Pied Flycatcher** | Fic_hyp | 1.1914 | 0.0007 | 2 | 6002 |
| ***Fringilla coelebs*** | **Common Chaffinch** | Fri_coe | 0.5927 | -0.0052 | 1 | 1018 |
| ***Garrulus glandarius*** | **Eurasian Jay** | Gar_gla | 0.3472 | 0.0205 | 1 | 0 |
| ***Lophophanes cristatus*** | **Crested Tit** | Lop_cris | 1.7711 | -0.0075 | 1 | 0 |
| ***Loxia curvirostra*** | **Red Crossbill** | Lox_cur | 1.6954 | -0.0032 | 0 | 0 |
| ***Muscicapa striata*** | **Spotted Flycatcher** | Mus_str | 0.8171 | 0.006 | 2 | 6975 |
| ***Parus major*** | **Great Tit** | Par_maj | 0.3701 | 0.0076 | 1 | 0 |
| ***Periparus ater*** | **Coal Tit** | Per_ate | 1.3347 | -0.0026 | 1 | 98 |
| ***Phoenicurus phoenicurus*** | **Common Redstart** | Pho_pho | 0.5913 | 0.0213 | 1 | 4926 |
| ***Phylloscopus collybita*** | **Common Chiffchaff** | Phy_col | 0.3367 | 0.0051 | 2 | 3563 |
| ***Phylloscopus sibilatrix*** | **Wood Warbler** | Phy_sib | 1.2979 | -0.0204 | 2 | 5753 |
| ***Phylloscopus trochilus*** | **Willow Warbler** | Phy_tro | 0.7514 | -0.0167 | 2 | 7301 |
| ***Picus canus*** | **Grey-headed Woodpecker** | Pic_can | 0.6177 | 0.0779 | 2 | 0 |
| ***Picus viridis*** | **European Green Woodpecker** | Pic_vir | 0.3581 | 0.0489 | 2 | 0 |
| ***Poecile montanus*** | **Willow Tit** | Poe_mon | 0.4953 | 0.0135 | 1 | 0 |
| ***Poecile palustris*** | **Marsh Tit** | Poe_pal | 0.6359 | 0.0145 | 1 | 0 |
| ***Prunella modularis*** | **Dunnock** | Pru_mod | 0.8155 | -0.0201 | 1 | 1555 |
| ***Pyrrhula pyrrhula*** | **Eurasian Bullfinch** | Pyr_pyr | 1.5746 | -0.0097 | 0 | 427 |
| ***Regulus ignicapilla*** | **Common Firecrest** | Reg_ign | 1.5898 | 0.0058 | 2 | 468 |
| ***Regulus regulus*** | **Goldcrest** | Reg_reg | 1.5068 | -0.0399 | 2 | 376 |
| ***Sitta europaea*** | **Eurasian Nuthatch** | Sit_eur | 0.691 | 0.0234 | 1 | 0 |
| ***Spinus spinus*** | **Eurasian Siskin** | Spi_spi | 1.3317 | -0.0067 | 1 | 1275 |
| ***Sturnus vulgaris*** | **Common Starling** | Stu_vul | 0.7176 | 0.0245 | 1 | 1286 |
| ***Sylvia atricapilla*** | **Eurasian Blackcap** | Syl_atr | 0.3198 | 0.0278 | 1 | 3444 |
| ***Troglodytes troglodytes*** | **Eurasian Wren** | Tro_tro | 0.9023 | 0.0062 | 2 | 458 |
| ***Turdus merula*** | **Common Blackbird** | Tur_mer | 0.3409 | 0.0191 | 1 | 772 |
| ***Turdus philomelos*** | **Song Thrush** | Tur_phi | 0.3775 | 0.0158 | 1 | 1981 |
| ***Turdus viscivorus*** | **Mistle Thrush** | Tur_vis | 0.9515 | 0.0141 | 1 | 1475 |
